# Spatiotemporal Expression Control Correlates with Intragenic Scaffold Matrix Attachment Regions (S/MARs) in Arabidopsis thaliana

**DOI:** 10.1371/journal.pcbi.0020021

**Published:** 2006-03-31

**Authors:** Igor V Tetko, Georg Haberer, Stephen Rudd, Blake Meyers, Hans-Werner Mewes, Klaus F. X Mayer

**Affiliations:** 1 GSF National Research Center for Environment and Health, MIPS, Institute for Bioinformatics, Neuherberg, Germany; 2 Bioinformatics Group, Turku Centre for Biotechnology, Tykistokatu, Turku, Finland; 3 Department of Genome-Oriented Bioinformatics, Wissenschaftszentrum Weihenstephan, Technische Universität München, Freising, Germany; 4 Department of Plant and Soil Sciences, Delaware Biotechnology Institute, Newark, New Jersey, United States of America; University of California San Diego, United States of America

## Abstract

Scaffold/matrix attachment regions (S/MARs) are essential for structural organization of the chromatin within the nucleus and serve as anchors of chromatin loop domains. A significant fraction of genes in Arabidopsis thaliana contains intragenic S/MAR elements and a significant correlation of S/MAR presence and overall expression strength has been demonstrated. In this study, we undertook a genome scale analysis of expression level and spatiotemporal expression differences in correlation with the presence or absence of genic S/MAR elements. We demonstrate that genes containing intragenic S/MARs are prone to pronounced spatiotemporal expression regulation. This characteristic is found to be even more pronounced for transcription factor genes. Our observations illustrate the importance of S/MARs in transcriptional regulation and the role of chromatin structural characteristics for gene regulation. Our findings open new perspectives for the understanding of tissue- and organ-specific regulation of gene expression.

## Synopsis

Scaffold/matrix attachment regions (S/MARs) are AT-rich DNA sequences that mediate structural organization of the chromatin within the nucleus. These elements constitute anchor points of the DNA for the chromatin scaffold and serve to organize the chromatin into structural domains. Studies on individual genes led to the conclusion that the dynamic and complex organization of the chromatin mediated by S/MAR elements plays an important role in the regulation of gene expression. In addition to intergenic S/MARs, which likely exert import insulator effects, more than 2,000 intragenic S/MARs have been shown to be present within the *Arabidopsis* genome. In this study, the authors set out to analyze the effects of these intragenic S/MAR elements on the regulation of the genes affected. Making use of exhaustive and multidimensional expression datasets available for *Arabidopsis,* the authors analyzed overall expression differences and correlation of intragenic S/MARs with spatiotemporal expression of genes. On a genome scale, pronounced tissue- and organ-specific and developmental expression patterns of S/MAR-containing genes have been detected. Notably, transcription factor genes contain a significant higher portion of S/MARs. The pronounced difference in expression characteristics of S/MAR-containing genes emphasizes their functional importance and the importance of structural chromosomal characteristics for gene regulation in plants as well as within other eukaryotes.

## Introduction

Scaffold/matrix attachment regions (S/MARs) are structural elements of eukaryotic cells [1]. S/MARs are required for the compaction and anchoring of chromatin to the nuclear framework. These regions are approximately 300 base pairs to several kilobases in length, and they are present in all higher eukaryotes, including mammals and plants [2,3]. S/MARs are defined as DNA elements that specifically bind to the nuclear matrix and as DNA fragments that copurify with the nuclear matrix [4]. Involvement of S/MARs in the regulation of gene activity and in the stabilization of expression has been shown for individual genes and S/MARs [5]. For vertebrates, a striking overlap of conserved noncoding elements and S/MAR functionality has been reported [6]. Glazko and coworkers reported that an excess of conserved vertebrate S/MAR regions was detected in intergenic regions preceding the 5′ end of genes, suggesting that these attachment regions might be involved in transcriptional control. These conclusions made for vertebrates are supported by our previous analysis of the correlation of S/MAR elements and expression levels in Arabidopsis thaliana. S/MAR-containing genes (S/MAR+ genes) have been shown to reach overall significantly lower expression levels compared to genes not associated with S/MARs, or lacking S/MARs (S/MAR− genes) [7]. Thus, intragenic S/MARs show a negative correlation with the transcriptional level of the S/MAR-containing gene and therefore may be involved in regulation of gene expression.

It has been hypothesized that, apart from transcriptional control mediated by specific transcription factors (TFs) and their respective *cis*-regulatory promoter binding sites, higher-level spatial and temporal chromosome topology within the nucleus and its association with the nuclear matrix exert important regulatory functions. For individual S/MARs, tissue and temporal regulatory roles are well established [1,8,9]. However, thus far, for no organism has a comprehensive and genome scale analysis been undertaken to investigate the implications of S/MAR presence within genes with respect to transcriptional activity. With the availability of a high-quality genome template for *Arabidopsis* and the localization of S/MARs on the complete genome [7] as well as the availability of high quality expression data [10−13], it has become feasible to address questions regarding the influence of intragenic S/MARs on spatiotemporal regulation of transcription. In our analysis, we made use of the available expression data that measure expression within different tissues, organs, and developmental stages. Our results provide evidence that the presence of an intragenic S/MAR not only correlates with the expression levels of genes but also shows a pronounced specificity for tissues, organs, and developmental phases. This allows the conclusion that intragenic S/MARs not only serve as static organizers of nuclear and chromosomal structure but also reflect the presence of potentially dynamic DNA elements that exert important regulatory functions on the expression of individual genes.

##  Results

In our analysis, we used S/MARs that were detected as described in our previous analysis [7]. Within this study we showed that S/MAR+ genes containing S/MAR elements have an overall lower expression level. This has been measured by EST associations as a proxy for expression strength as well as by MPSS (Massive Parallel Sequencing Signature). The MPSS technology produces short sequence signatures produced from a defined position within an mRNA, and the relative abundance of these signatures in a given library represents a quantitative estimate of expression of that gene. To this end, no distinction between different organ and tissue expression values has been made, and potential correlations between tissue and pattern distributions and the presence/absence of S/MARs have not been analyzed.

### High-Resolution Expression Datasets Enable High-Resolution Study of S/MAR Effects on Transcriptional Properties

For this study, we used expression datasets that were generated with different experimental foci and by using different technical platforms. The first expression dataset was obtained by the MPSS technology [7,12,13]. The individual MPSS tags were mapped onto the *Arabidopsis* genome and unambiguous MPSS tags were selected (see Materials and Methods).

In addition, a root expression dataset, termed digital in situ data, has been used for the analysis. It was derived from a high-resolution spatial and temporal expression profile throughout the *Arabidopsis* root [10]. These data represent a global expression map of the *Arabidopsis* root for 22,000 genes, with measurements taken within six different tissues or tissue combinations (stele, endodermis, endodermis plus cortex, epidermal atrichoblast cells, and lateral root cap), as well as three time points of development stages defined by their distance from the apical root meristem (Table 1).

**Table 1:**
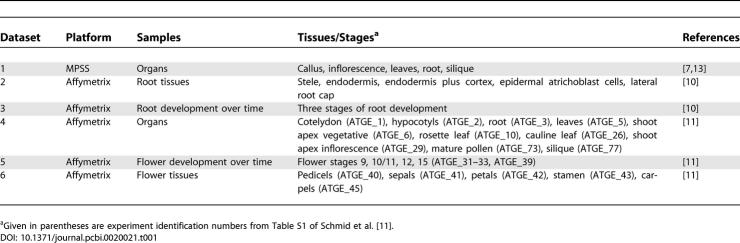
Experimental Datasets Used in the Analysis

Finally, we also used an expression dataset derived from the AtGenExpress project, which is composed of 79 different experiments covering a wide range of developmental stages, organs, and organ systems [11]. We selected three datasets representing the expression within ten different organs, five flower tissues, and five stages of flower development (Table 1).

The root dataset and the AtGenExpress project are based on the ATH1 Affymetrix platform. 

### S/MAR+ Genes Are Less Expressed Irrespective of the Tissue and Organ

**Figure 1:**
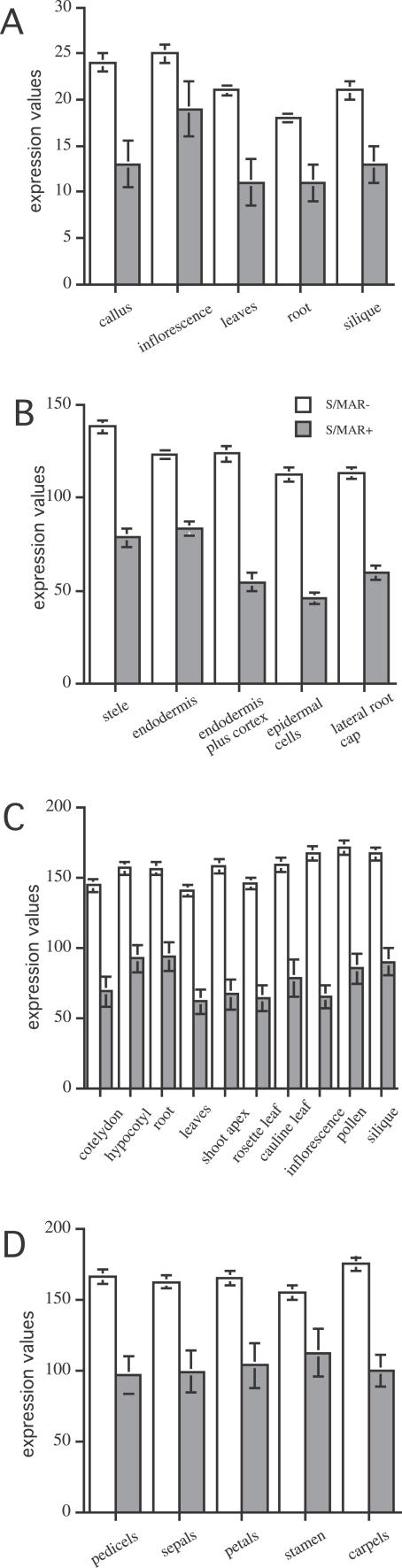
Median Expression Values of S/MAR− and S/MAR+ Genes in Different Organs, Root Tissues, and Flower Tissues MPSS data recorded in five different organs are shown (Table 1, dataset 1) (A); for Affymetrix-based measurements (B–D), median values for five root tissues (Table 1, dataset 2) (B), ten organs (Table 1, dataset 4) (C), and five flower tissues (Table 1, dataset 6) (D) are given. For MPSS-based experiments (A), tpm are indicated; for experiments based on the Affymetrix platform (B–D), Affymetrix expression values are plotted. The 5% confidence intervals calculated using bootstrap set for all values are shown.

We calculated median expression values for datasets with measurements for organs and tissues (Table 1, datasets 1, 2, 4, and 6). Figure 1 shows that S/MAR+ genes have significantly lower expression values for all experiments. The ratio of expression of S/MAR− to S/MAR+ genes was in the range of 1.6:2, and the results were consistent between different experiments and platforms. In contrast to the ratios, the maximal expression values of each set of experiments were not significantly different for S/MAR+ and S/MAR− gene sets with the exception of the root dataset (Table 2, dataset 2).

**Table 2:**
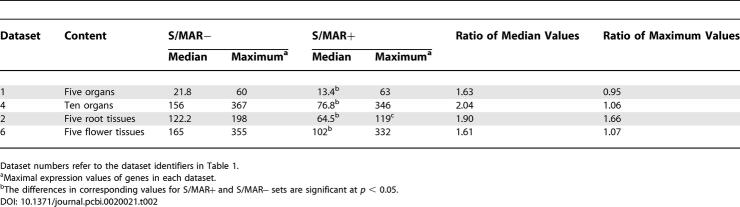
Expression Values of S/MAR− and S/MAR+ Genes for Different Datasets

### S/MAR+ Genes Are More Likely to Be Differentially Expressed

To ask whether S/MAR+ genes differ solely by overall transcription levels or whether the observed lower expression level is caused by a more pronounced differential expression in organs and tissues, we introduced the Differential EXpression Profile index (DEXP). The DEXP value corresponds to the relative expression level of genes in a given tissue compared to their maximum expression. Thus, the DEXP measures a different characteristic of the data, i.e., their skewness and tendency to be expressed within only some particular tissues, organs, or treatments. High DEXP values, those close to 1, are indicative of genes expressed in all tissues, organs, and developmental stages at similar levels. In contrast, genes with low DEXP values are preferentially expressed in one or very few experiments and thus have pronounced and confined expression domains.

**Figure 2:**
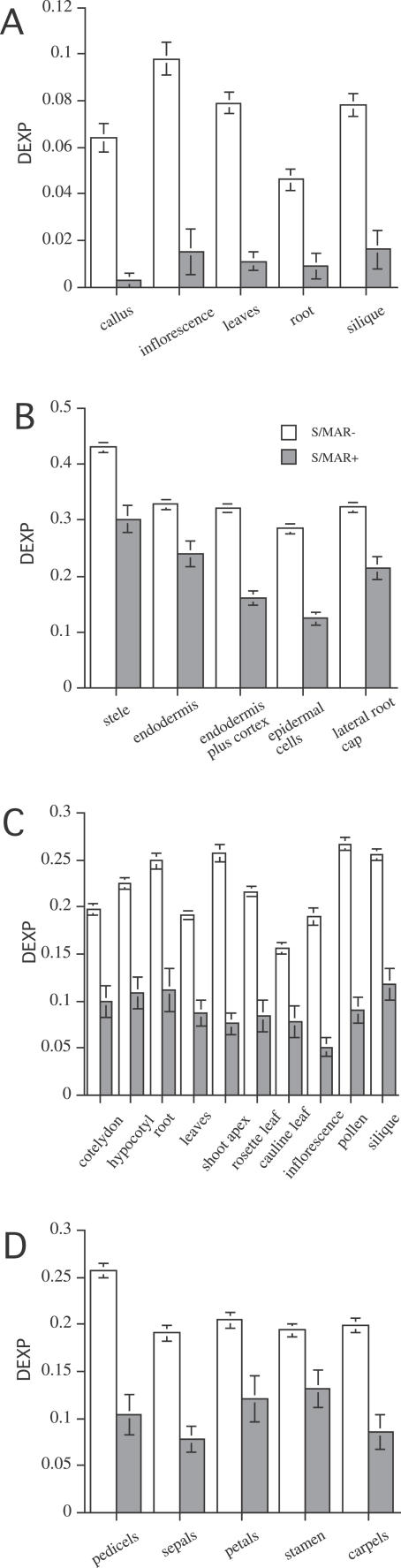
DEXP Values of S/MAR− and S/MAR+ Genes for Different Organs and Tissues (A) DEXP for MPSS data recorded in five different organs (Table 1, dataset 1). (B) DEXP for five root tissues (Table 1, dataset 2). (C) DEXP for ten organs (Table 1, dataset 4). (D) DEXP for five flower tissues (Table 1, dataset 6). There are different scales for different experiments. The 5% confidence intervals are shown as error bars.

For the tissues and organs analyzed, S/MAR+ genes show a significantly lower DEXP value compared to S/MAR− genes (Figure 2). S/MAR+ genes therefore tend to be confined to specific tissues or organs. While their median expression values over all tissues are lower, the maximum expression values of S/MAR+ genes are in a similar range as for S/MAR− genes. The MPSS data produced the smallest DEXPs for the S/MAR+ genes. This result may indicate a lower level of noise in the data generated by this technology as compared to the Affymetrix technology [13,14]. A higher level of noise may raise expression values of nonexpressed genes and thus increase the DEXP values. In addition, the MPSS method measures the absolute expression values of gene expression, while for Affymetrix-based measurements only relative expression values are used. Furthermore, the results measured using Affymetrix can be also sensitive to cross-hybridization effects [15], which may decrease the differences between highly expressed and nonexpressed genes.

To perform a direct comparison for MPSS- versus Affymetrix-based expression measurements in different organs (Table 1, datasets 1 and 4), we calculated the DEXP values for the four organs common across both datasets, i.e., inflorescence, leaves, root, and silique. In this analysis, the DEXP profiles for S/MAR+ and S/MAR− genes were similar for both data types, as indicated in Figure S1, but had different absolute values. Nevertheless, although the absolute expression and DEXP values are not directly comparable between both platforms, significant differences in gene expression between S/MAR+ and S/MAR− genes within each experiment are consistent among all experiments and platforms.

In summary, S/MAR+ genes had significantly lower DEXP values compared to S/MAR− genes. The pronounced differential expression produced a lower median expression value for S/MAR+ genes, while the maximum expression of S/MAR+ genes were in the same range as S/MAR− genes. These results suggest that intragenic S/MARs may be involved in tissue/organ-specific regulation of expression.

### Developmental Profiles of S/MAR+ Genes

**Figure 3:**
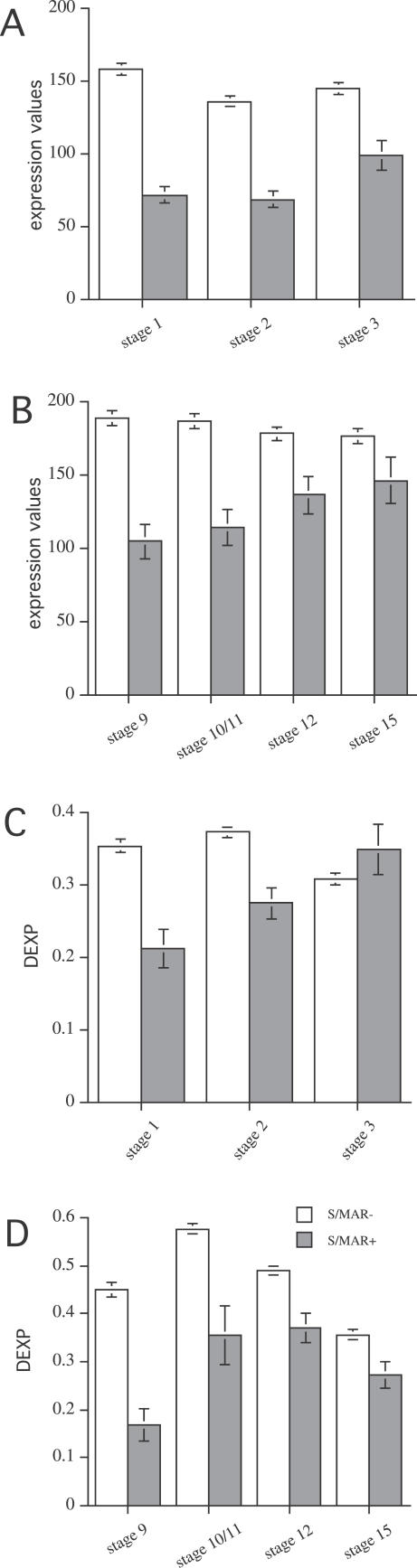
Median Expression and DEXP Values for Different Developmental Stages of Root and Flower The median expression values and the DEXP values for three different developmental stages of the root (A and C) and four developmental stages of the flower (B and D) are given. The respective stage classifier is given on the *x*-axis. The differences in DEXP values of S/MAR+ and S/MAR− genes decrease with the increasing age of the tissues. The 5% confidence intervals for all values are shown.

As the presence of genic S/MARs showed a pronounced influence on the specificity of expression, we were interested in whether similar effects can be detected for developmental time courses. To address this question, we used the expression data available for three stages of root development (Table 1, dataset 3) and five stages of floral development (Table 1, dataset 5). We again analyzed the mean expression values as well as the differential expression of S/MAR+ versus S/MAR− genes for different developmental stages of roots and flowers, respectively (Figure 3). S/MAR+ genes had significantly lower median expression values and DEXPs than did S/MAR− genes for all stages, with the exception of stage 3 within the root dataset. These observations are indicative of a regulatory role exerted by S/MAR elements during the development of roots and flowers. The differences in DEXP values and median expression values between both groups of genes decreased with increasing developmental stages of the organ. Thus, in the final stages of development and differentiation of the organs, the regulatory effect of intragenic S/MARs declines and median and differential expressions of S/MAR+ and S/MAR− genes become similar.

### TFs Contain Disproportionate Amounts of S/MARs and Are Highly Differentially Expressed

**Figure 4:**
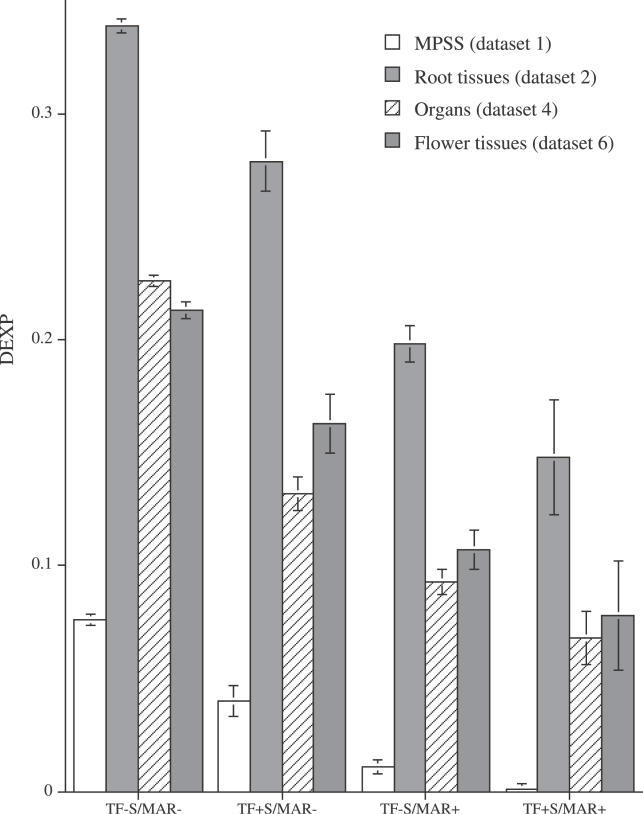
DEXP Values for S/MAR+ and S/MAR− TF Genes for Different Datasets The 5% confidence intervals for all values are shown.

TFs are key regulators of transcriptional activity of genes. With the pronounced differences in expression observed for developing tissues and between different organs, we examined the extent of S/MAR presence within TF genes and asked whether the pronounced temporal and spatial differences observed for S/MAR+ genes can also be found for TFs. This analysis used all 1,611 TF genes listed in the Arabidopsis thaliana transcription factor database (http://arabidopsis.med.ohio-state.edu/AtTFDB) [16]. Analysis of these TFs showed that 240 TF genes (15%) contained S/MAR regions. This proportion is one-half times higher compared to the overall percentage of 9.8% S/MAR+ genes in the genome (*p* < 10^−8^ according to binomial test). We analyzed differential expression of genes and TFs with and without S/MARs using the DEXP. We designate TF genes that also contain an S/MAR as TF+ S/MAR+, other TF genes as TF+ S/MAR−, and remaining S/MAR+ genes as TF− S/MAR+ (Figure 4).

For all analyzed tissue and organ datasets, we detected pronounced differences among the distinct categories of genes. The DEXP values were significantly lower for TF genes compared to non-TF genes. This result is in agreement with well-established knowledge that TF genes tend to be differentially expressed [17]. However, S/MAR+ genes had significantly lower DEXP values than TF genes and thus display even higher tissue- and organ-specific expression compared to TFs. The TF genes containing S/MARs showed the lowest DEXP values for all organ and tissue datasets and thus had the highest probability to have tissue- or organ-specific expression among all analyzed categories of genes. In summary, we observed a synergistic effect for tissue- and organ-specific expression for TF genes and the presence of intragenic S/MARs.

An analysis of datasets of different developmental stages in roots and flowers (Table 1, datasets 3 and 5) gave similar results for expression of TF and S/MAR+ genes (Figure S2). As in the case of tissue and organ specificity, the TF− S/MAR+ genes had significantly lower DEXP values compared to TF− S/MAR− genes. The DEXP values of TF genes containing S/MAR elements show a pronounced variance, and no significant difference in expression of this group of genes compared to TF or S/MAR+ genes was detected for expression data from different root developmental stages. For the flower development (Table 1, dataset 5), DEXP values of TF+ S/MAR+ genes were significantly lower compared to TF genes but not compared to the S/MAR+ genes.

### S/MARs Are Significantly Overrepresented within Specific TF Families

TF genes available from the Arabidopsis thaliana transcription factor database have been subclassified into 42 families [16]. We analyzed whether specific TF families are enriched for S/MAR+. We found three notable families (Table 3). Genes in the homeobox family, the MADS box family, and the basic helix-loop-helix family contain overrepresented amounts of S/MARs (30.7%, 28.2%, and 22.8% S/MAR+, respectively; *p*-values <0.00005 to <0.001; Table 3).

**Table 3:**
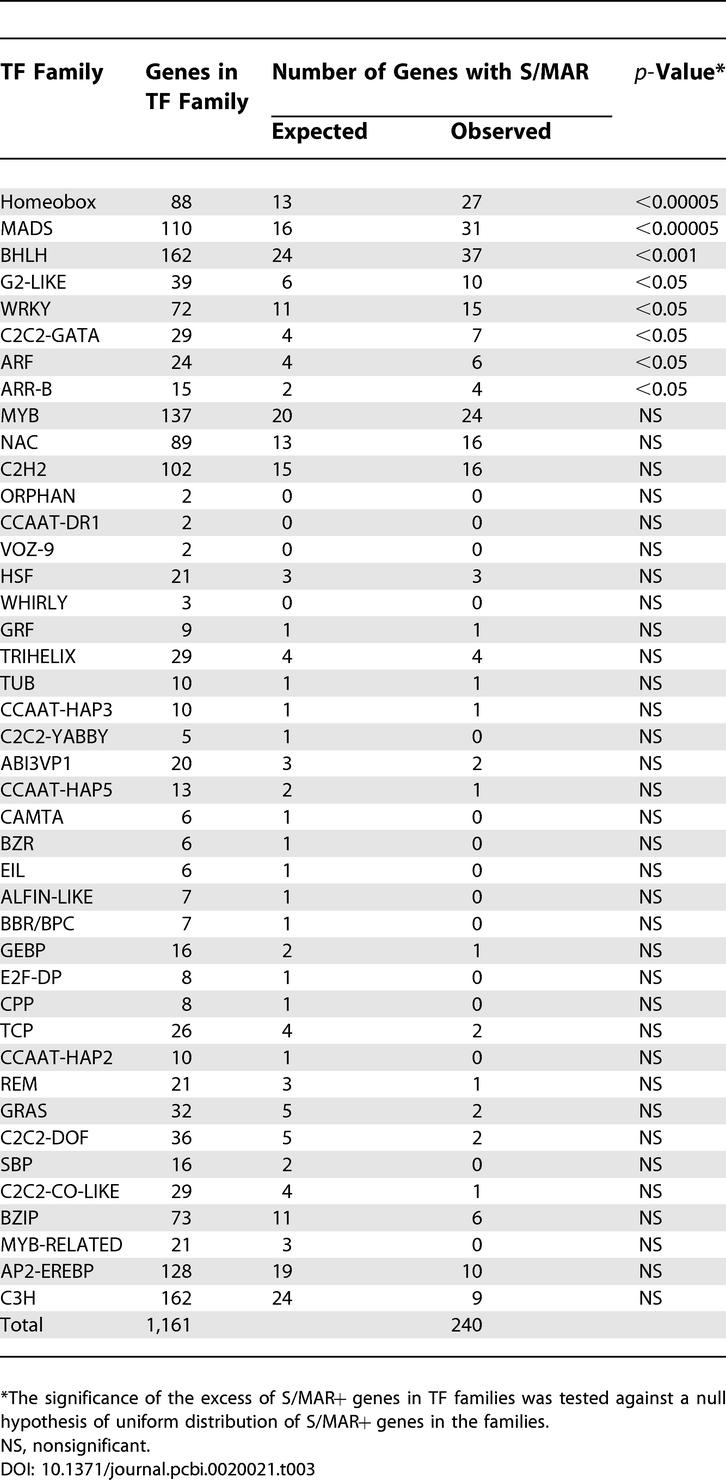
Distribution of TF Genes Containing S/MARs within Different TF Families

These groups contain numerous well-studied TFs with important roles in the development and during the life cycle of plants for which specific expression localization has been demonstrated. Examples include the *WUSCHEL* (WUS) [18], *SHOOTMERISTEMLESS* (STM) [19], and *BELL* (BEL1) [20] genes within the homeobox class and the *AGAMOUS* (AG), *APETALA 1* and *3* (AP1, AP3), and *SEPALATA 1* and *3* (SEP1 and SEP3) in the MADS box class [21−23]. A complete listing of S/MAR-containing TFs is provided in Table S1.

### The Degree of Differential Expression Varies for Different Intragenic S/MAR Localizations

**Figure 5:**
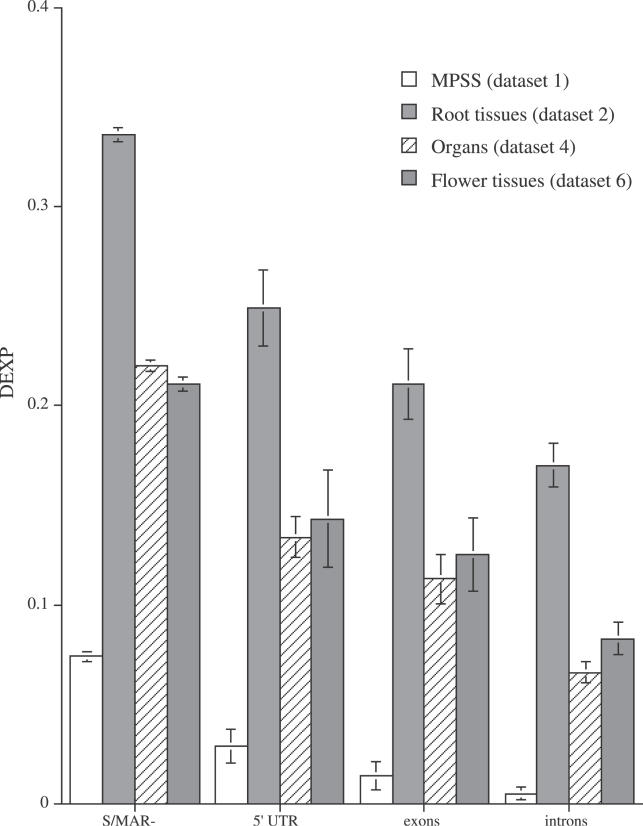
DEXP Values for S/MAR− Genes and as a Function of the Position of the S/MAR within S/MAR+ Genes The 5% confidence intervals are shown.

For all analyses listed above, genes that contained S/MARs within the 5′ UTR, protein-coding exons, or introns were considered to be S/MAR+ genes. We assessed whether DEXP values vary with the position of an S/MAR element within the gene. As indicated at Figure 5, the DEXP values of S/MAR+ genes depend on the position of the attachment region. Genes containing S/MARs within introns have significantly lower DEXP values compared to genes with S/MAR regions in the 5′ UTR or exons. These findings are consistent for both Affymetrix- and MPSS-based MPSS datasets.

## Discussion

To gain insight into the role and correlation of the presence of S/MAR elements on the spatiotemporal control of gene expression, we analyzed genomewide, multidimensional expression data for A. thaliana. In our previous analysis, we reported the detection of 21,705 potential S/MAR elements, among which 2,135 have been localized within genes [7]. In functional tests, individual S/MARs have been demonstrated to act as insulators, by protecting a loop from the effects of the neighboring chromatin or associated enhancer sequences [24,25], and their action has been demonstrated to be highly context dependent [26]. Numerous studies demonstrated the influence of bordering, insulator-type elements as well as the complex interplay of intronic enhancers and bordering S/MAR elements in the transcriptional control of individual genes [25−27] (and references therein).

We addressed the questions of whether and to which extent intragenic S/MARs affect transcriptional control in A. thaliana. For this analysis, we made use of the exhaustive expression datasets available for different tissues, organs, and life phases of *Arabidopsis* [10,11,13]. The expression values of S/MAR+ genes and S/MAR− genes were analyzed for their expression characteristics. We identified several important features of S/MAR+ genes.

The analysis of expression based on MPSS data, digital in situ expression data for different root tissues, and Affymetrix expression data covering a broad range of tissues, organs, and development stages all indicated that S/MAR+ genes were significantly lower expressed compared to S/MAR− genes. This is in line with previous results that were based on only a global expression level [7]. In addition, our analysis demonstrates that the global downregulatory effect of genic S/MARs can be detected in all analyzed organs and tissues. However, maximal expression levels were similar between S/MAR+ and S/MAR− genes.

We introduced the DEXP to quantitatively differentiate between genes showing pronounced expression peaks for individual tissues and organs from genes that are widely expressed. A low DEXP value is indicative of a pronounced organ- or tissue-specific expression pattern, whereas a high DEXP value characterizes genes that are expressed at similar levels over a wider range of tissues and organs and thus show no or less-pronounced organ and tissue specificity. We found that S/MAR+ genes had significantly lower DEXP values compared to those of S/MAR− genes. Thus, the S/MAR+ genes were preferentially expressed in only one of the analyzed conditions and their expression was tissue and organ specific and dependent on the developmental stage. We found that a high proportion of TF genes contain S/MARs. The overall DEXP value found for TF genes was lower than that for the other classes of genes. However, S/MAR presence within TF genes leads to a pronounced decrease in the DEXP value, suggesting a more pronounced spatiotemporal regulation of TF+ S/MAR+ genes.

The differential expression in S/MAR+ genes was visible in the decreased DEXP and was related to the position of the S/MAR within the gene. Genes that contain S/MARs within introns had significantly lower DEXP values compared to genes that contain S/MARs within 5′ UTRs or exons. Moreover, the likelihood of detecting S/MAR regions within the introns was also about two times higher compared to within exons [7]. These findings are consistent with the important role of intronic S/MARs in the regulation of individual genes such as the *immunoglobulin heavy-chain* locus *(Igh)* [28]. In summary, our findings indicate that the presence of S/MARs within introns is the dominating mechanism for S/MAR-mediated tissue-, organ-, and development-dependent transcriptional regulation in plants.

The expression values measured by diverse technologies (i.e., MPSS and Affymetrix) and values obtained from different laboratories resulted in dissimilar expression and DEXP values for S/MAR+ genes (Figures 1–5). Direct comparison of the various datasets was not feasible; therefore, our analyses were based on comparisons of S/MAR+ and S/MAR− genes between individual, comparable sets of experiments. The significant differences between both groups of genes were consistently observed across the various datasets.

Our results are supportive of an important role of S/MARs in spatiotemporal transcriptional regulation. We found pronounced differences for S/MAR+ and S/MAR− genes in all organ-derived datasets we analyzed, for datasets reflecting the transcriptional state in different tissues, and for data reflecting different developmental time points. To the best of our knowledge, this is the first observation of strong and significant correlations of the presence of S/MARs and the spatiotemporal control of gene expression on a genome scale.

However, individual genes and S/MARs associated with them have been studied and biochemically characterized. For example, an interaction of the chicken S/MAR binding proteins SATB1 and SATB2 with S/MAR regions provided tissue-specific expression of gene regulation in mouse [8,29,30]. SATB1 acts as a cell-type–specific genome organizer and gene regulator essential for T-cell differentiation and activation. SATB1 thereby represses numerous genes, and biochemical data indicate that repression is mediated by histone H3 deacetylation at Lys9 and Lys14 [31−34]. Repression via histone deacetylation through an S/MAR associated with *SATB1* has been analyzed in detail [31,35]. The biochemical basis of S/MAR action on a genomic scale is as yet unknown and will be the subject of future analyses. However, in *Arabidopsis* the regulation of transcriptional activity through modification of histones is well established, and this can lead to chromatin compaction through heterochromatin formation [36]. More recently, an important role of histone acetylation and chromatin remodeling in mediating gene expression based on positional cues has been demonstrated in *Arabidopsis* roots and leaves [37,38].

Recent studies propose that morphological and functional heterogeneity of the nucleus is generated by the presence of distinct nuclear compartments [39]. Such observations have led to the development of advanced concepts of the nuclear architecture and the structural integration of chromosomes within the nucleus. An important influence of the nuclear organization on gene activity has been hypothesized. Several recent studies indicate a tight correlation of chromosome territory (CT) structure and transcriptional activity [40,41]. CT structure has been hypothesized to be important to render a transcriptionally poised state prior to activation. In addition, the CT structure has been suggested as an important mechanism in cell-type–specific transcriptional activation or repression [9]. Thus, there are higher-order levels of transcriptional control in addition to *cis-*regulation by TFs.

The existence of different types of S/MARs as structural and functional elements has been proposed [42−44]. In addition, it has been shown recently that multiple-copy S/MARs are selected and used as nuclear matrix anchors in a discriminatory manner, even though they all contained identical primary sequences [45]. It has been hypothesized that the underlying selection process is mediated by S/MAR availability influenced by position on the DNA, binding strength, and/or copy number. Although S/MARs function as the mediators of loop attachment, they might be used in a selective and dynamic fashion. Consequently, S/MAR anchors are necessary but not sufficient for chromatin loops to form. Some of the predicted S/MAR attached regions could serve as regulatory elements and display dynamic characteristics, while others will not have this function and fulfill structural roles. Therefore, potentially only a fraction of S/MAR regions could be involved in tissue-specific gene regulation, while others might potentially fulfill only structural roles [1,42−44]. Our results suggest that intragenic S/MARs are likely to be the functional elements proposed in the aforementioned studies. The question of whether there are some preferences for intergenic S/MARs to be functional or structural elements will require further analysis.

### Conclusions

We performed a genome-scale comparative analysis of expression patterns of genes containing predicted S/MAR attachment regions in A. thaliana using three different expression datasets generated on two different platforms. All analyses provided consistent results. Genes containing predicted S/MAR regions have significantly lower DEXP values and are likely to be expressed in one tissue/organ or developmental phase. As a consequence of a difference in DEXP values, S/MAR+ genes have lower expression values compared to S/MAR− genes. Thus, S/MAR+ genes serve or are used as triggers for the tissue, organ, and developmental specificity in *Arabidopsis.* Approximately 15% of TF genes contain predicted attachment regions. Moreover, these TF+ S/MAR+ genes have significantly lower DEXP values compared to other TF genes as well as other S/MAR+ genes. This subset of genes may correspond to TFs directly involved in tissue-, organ-, and development-specific patterns of gene expression.

## Materials and Methods

### Prediction of S/MAR+ genes.

In a previous analysis, we reported the genomewide analysis and identification of S/MARs within the *Arabidopsis* genome [7]. The S/MAR prediction was performed using the SMARTest program [46] (http://www.genomatix.org). SMARTest is based on a library of S/MAR-associated, AT-rich patterns derived from comparative sequence analysis of experimentally defined S/MAR sequences. As reported previously [7], the training set contained 16 plant-derived S/MARs (seven from *Arabidopsis*). SMARTest has been applied using default *Arabidopsis* settings. A sensitivity of 38% and a specificity of 68% have been demonstrated [46]. A recent evaluation of different S/MAR finders confirmed SMARTest as outperforming with respect to specificity [47]. Within our previous analysis, we reported the identification of a total of 21,705 S/MARs across the genome [7]. Two thousand one hundred thirty-five S/MARs have been found to be located within genes (9.8%) as defined by a localization of the respective S/MAR within either the coding regions or introns of a gene [7]. The coordinates delimiting the chromosomal location of each of the candidate S/MARs were anchored to the pseudomolecules as described elsewhere [7]. For this analysis, we used data from our previous study and included additional 590 genes containing S/MAR regions within the 5′ UTR regions (see Table S1 for a full list of S/MAR+ genes).

### Expression datasets.

Expression data from three independent sources have been used. Details are given in the text as well as within Table 1.

### MPSS dataset.

The data from MPSS experiments [12,13] were used and described in detail in our previous analyses [7,48]. MPSS represents a powerful means for the quantitative measurement of gene expression [14], and it can identify and analyze the level of expression of all genes in a sample by counting the number of individual mRNA molecules. MPSS provides a quantitative estimate of expression as opposed to the relative estimates derived from hybridization signal intensities on microarrays.

The number of MPSS tags per gene was in the range of one to approximately ten. Some of the tags were not unique and could be mapped to several A. thaliana genes simultaneously. After careful analysis, we selected 1,383 S/MAR+ and 13,804 S/MAR− genes that could be each unambiguously mapped to A. thaliana genes. For this analysis, we selected a subset of genes that had an MPSS value greater than 10 transcripts per million (tpm) units for at least one of the measurements [7]. This filtering removed genes with low expression values that may not allow us to differentiate between expressed and nonexpressed genes. The resulting subset of MPSS-tagged S/MAR+ genes contained 952 genes as well as 10,340 S/MAR− genes (8.4%). We used the data corresponding to five organs: callus, inflorescence, leaves, root, and silique (Table 1).

### Microarray expression datasets.

The microarray datasets were derived from two recent works [10,11].

The root expression dataset used for the analysis consisted of a high-resolution spatial and temporal expression profile throughout the *Arabidopsis* root [10]. The expression data, termed digital in situ data, reflect gene expression among cell types and tissues and along a developmental gradient. The regular radial organization of the root and the continuous development facilitate the analysis of gene expression on a spatiotemporal axis. The data included a global map of gene expression for 22,000 genes measured by Affymetrix microarrays. The gene expressions were measured in six different locations (stele, endodermis, endodermis plus cortex, epidermal atrichoblast cells, and lateral root cap) and three time development stages defined by their distance from the apical meristem (Table 1). The data were downloaded from http://www.arexdb.org.

Finally, an expression dataset derived from the AtGenExpress project, which comprised 79 different experiments, has been used. The experiments cover a wide range of developmental stages, organs, and organ systems of *Arabidopsis* [11]. We selected three datasets covering expression of A. thaliana in ten different organs, five flower tissues, and five stages of flower developments. Only experiments that involved similar genetic background (wild-type), the same substrate (soil), and the same photoperiod (continuous light) were considered (Table 1).

An analysis of expression values indicated a considerable increase in the slope of the number of gene expressions with values below 15 to 20 units for both AtGenExpress and root datasets. This change may correspond to the increase in the noise level for such low expression values; i.e., this value can be taken as a threshold of sensitivity of the method. We decided to filter out all genes that had a maximal expression value over all tissues less than 30 units, i.e., similar to the 10-tpm threshold used for the MPSS data. After filtering, the expression data for the root dataset [10] contained 1,907 S/MAR+ and 18,311 S/MAR− genes. The data derived from AtGenExpress [11] contained 1,602 S/MAR+ and 16,648 S/MAR− genes. Thus, the genes predicted to be S/MAR+ accounted for about 8% to 9% of all genes for three analyzed datasets.

### Median values and significance test.

We used median expression values and DEXP indices for the comparison of different datasets. A two-tailed bootstrap test with 10,000 replicates was used to assess statistical significance. The 5% confidence intervals are depicted within all figures for all results.

### DEXP.

This index was introduced to measure the skew of the gene expression across different tissues and organs. The index measures residual expression of a gene in tissues and organs compared to its maximum expression. When analyzing several expressions, *i = 1,…,m* of gene *j,* we first determined a tissue or organ, *k,* for which a maximum expression value of the gene was observed, *E_j_^k^.* The index values were calculated as median values of the square of ratios of the gene expression at the target tissue to the tissue *k* with the maximum expression. This can be mathematically formulated as follows:


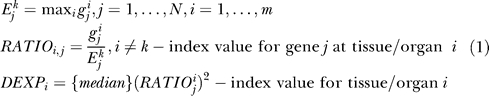


where *j* indicates the gene, *m* is number of tissues, and *N* is total number of the analyzed genes.

## Supporting Information

Figure S1 Median Expression Values and DEXP Values for Four Organs Measured by MPSS and Affymetrix Chips Median expression values (A and C) and DEXP values (B and D) for inflorescence. Leaves, root, and siliques are depicted. The 5% confidence intervals calculated using bootstrap set for all values are shown.
(59 KB DOC)

Figure S2 DEXP Values for S/MAR+ and S/MAR− TF Genes for Root and Flower Developmental Expression Datasets The 5% confidence intervals calculated using bootstrap set for all values are shown.
(25 KB DOC)

Table S1 List of SMAR+ and SMAR− *Arabidopsis* Genes, Their DEXP for Various Datasets, and TF Family Assignment (2.4 MB XLS)

## Abbreviations

CT: chromosome territory
DEXP: Differential EXpression Profile index
MPSS: Massive Parallel Signature Sequencing
S/MAR: scaffold/matrix attachment region
TF: transcription factor
tpm: transcripts per million
