# Correction: Spatiotemporal Expression Control Correlates with Intragenic Scaffold Matrix Attachment Regions (S/MARs) in Arabidopsis thaliana

**DOI:** 10.1371/journal.pcbi.0020067

**Published:** 2006-06-30

**Authors:** Igor V Tetko, Georg Haberer, Stephen Rudd, Blake C Meyers, Hans-Werner Mewes, Klaus F. X Mayer

In *PLoS Computational Biology,* vol 2, issue 3:  10.1371/journal.pcbi.0020021

Contributing author Blake Meyers' name and institution should appear:

Blake C. Meyers, Department of Plant and Soil Sciences, Delaware Biotechnology Institute, Newark, Delaware, United States of America

