# Supplementary material for: Spatiotemporal Expression Control Correlates with Intragenic Scaffold Matrix Attachment Regions (S/MARs) in Arabidopsis thaliana
Source: PLoS Comput Biol. 2006 Mar 31;2(3):e21. doi: 10.1371/journal.pcbi.0020021 (PMC1420657; doi:10.1371/journal.pcbi.0020021)
Supplement: Figure S1 — Median expression values (A and C) and DEXP values (B and D) for inflorescence. Leaves, root, and siliques are depicted. The 5% confidence intervals calculated using bootstrap set for all values are shown. (59 KB DOC) [file pcbi.0020021.sg001.doc]

Figure S1. Median expression values and DEXP values for four organs measured by MPSS (A, C) and Affymetrix chips (B, D). 5% confidence significant intervals calculated using bootstrap set for all values are shown.
