# Supplementary material for: Spatiotemporal Expression Control Correlates with Intragenic Scaffold Matrix Attachment Regions (S/MARs) in Arabidopsis thaliana
Source: PLoS Comput Biol. 2006 Mar 31;2(3):e21. doi: 10.1371/journal.pcbi.0020021 (PMC1420657; doi:10.1371/journal.pcbi.0020021)
Supplement: Figure S2 — The 5% confidence intervals calculated using bootstrap set for all values are shown. (25 KB DOC) [file pcbi.0020021.sg002.doc]

Figure S2. DEXP values for S/MAR containing and S/MAR non-containing transcription factor genes for root and flower developmental expression datsets. 5% confidence significant intervals calculated using bootstrap set for all values are shown.
